# Rapid PCR/ESI-MS-based molecular genotyping of *Staphylococcus aureus* from nasal swabs of emergency department patients

**DOI:** 10.1186/1471-2334-14-16

**Published:** 2014-01-09

**Authors:** Aleksandar Kecojevic, Ray Ranken, David J Ecker, Christian Massire, Rangarajan Sampath, Lawrence B Blyn, Yu-Hsiang Hsieh, Richard E Rothman, Charlotte A Gaydos

**Affiliations:** 1 Department of Community Health and Prevention, Drexel University School of Public Health, Philadelphia, PA, USA; 2 Department of Emergency Medicine, School of Medicine, Johns Hopkins University, Baltimore, MD, USA; 3 Ibis Biosciences, an Abbott Company, Carlsbad, CA, USA; 4 Department of Medicine, Division of Infectious Diseases, School of Medicine, Johns Hopkins University, Baltimore, MD, USA

## Abstract

**Background:**

A limitation of both culture-based and molecular methods of screening for staphylococcal infection is that current tests determine only the presence or absence of colonization with no information on the colonizing strain type. A technique that couples polymerase chain reaction to mass spectrometry (PCR/ESI-MS) has recently been developed and an assay validated to identify and genotype *S. aureus* and coagulase-negative staphylococci (CoNS).

**Methods:**

This study was conducted to determine the rates, risk factors, and molecular genotypes of colonizing *Staphylococcus aureus* in adult patients presenting to an inner-city academic emergency department. Participants completed a structured questionnaire to assess hospital and community risks for infection with methicillin-resistant *S. aureus* (MRSA). Nasal swabs were analyzed by PCR/ESI-MS to identify and genotype *S. aureus* and CoNS.

**Results:**

Of 200 patients evaluated, 59 were colonized with *S. aureus*; 27 of these were methicillin-resistant strains. Twenty-four of the 59 *S. aureus* carriers were co-colonized with a CoNS and 140 of the 200 patients were colonized exclusively with CoNS. The molecular genotypes of the 59 *S. aureus* strains were diverse; 21 unique molecular genotypes belonging to seven major clonal complexes were identified. Eighty-five of 200 patients carried strains with high-level mupirocin resistance. Of these eighty-five participants, 4 were colonized exclusively with *S. aureus*, 16 were co-colonized with *S. aureus* and CoNS, and 65 were colonized exclusively with CoNS.

**Conclusion:**

The prevalence of *S. aureus* and methicillin-resistant *S. aureus* colonization in a random sample of patients seeking care in Emergency Department was 29.5% and 13.5%, respectively. A substantial fraction of the *S. aureus*-colonized patients were co-colonized with CoNS and high-level mupirocin-resistant CoNS. Determining the molecular genotype of *S. aureus* during intake screening may prove valuable in the future if certain molecular genotypes become associated with increased infection risk.

## Background

*Staphylococcus aureus* is a major cause of healthcare-associated infections [1]. Although several epidemiological studies have provided information on the prevalence of methicillin-resistant *Staphylococcus aureus* (MRSA) colonization in different hospital settings [2-4] and in emergency department personnel [5,6], limited data exist on MRSA colonization rates for patients seen in emergency departments. The cost benefit of screening for MRSA colonization is not overwhelming and the subject is controversial [7]. A limitation of both culture-based and molecular methods of MRSA screening is that current tests determine only the presence or absence of colonization with no information on the colonizing strain type [8]. As only small subsets of MRSA clones have been responsible for the major epidemics in the United States [9] screening might have greater value if future studies demonstrate that certain clones are more likely to cause serious infections than others.

We have developed a rapid molecular surveillance assay based on PCR coupled to electrospray ionization mass spectrometry (PCR/ESI-MS) [10-12] that identifies and provides detailed information about *S. aureus* strains. A previous version of this assay has been shown to accurately genotype *S. aureus* strains and to identify the clonal complex of a series of isolates from the Centers for Disease Control (CDC). Excellent agreement with multilocus sequence typing (MLST) and pulsed-field gel electrophoresis (PFGE) results [13] was reached using in the PCR/ESI-MS assay a set of eight primer pairs that target the same genes as those for MLST. This set of primer pairs was designed to optimize the information content of the amplicons and distinguished 99% of 710 distinct *S. aureus* sequence types [13]. Using a second set of eight primer pairs, PCR/ESI-MS has also been used to characterize virulence factors, toxin-encoding genes and antibiotic resistance determinants on isolated colonies [14] and to detect MRSA in nasal swabs with sensitivity and specificity similar to culture methods [15]. In this work, elements from the previous studies were consolidated into a single PCR/ESI-MS research application to directly screen nasal swabs from sample of 200 patients who presented to an urban hospital emergency department.

## Methods

### Study setting

This prospective surveillance study of a convenience sample of adult patients was conducted in the Johns Hopkins Emergency Department, Maryland, from August 2008 to February 2009. The Johns Hopkins Hospital Emergency Department sees approximately 5,000 patients a month. An urgent care center (UCC, 12 beds) and a dedicated ‘Short-Stay’ emergency acute care unit (EACU, 17 beds) are also part of emergency department services and these patients were included in the study.

### Subject selection

All patients visiting the adult emergency department for evaluation and potential treatment were eligible for the study with the exception of prisoners and patients unable to understand the study requirements. The enrollment of patients was a sample of convenience during hours when the study coordinator was available and included patients seen Monday-Friday during the hours of 9:30 AM- 5:00 PM. We excluded patients with previously known risk factors for MRSA colonization [16-18] such as patients with fever greater than 100.4°F, abscesses, cellulitis, infected wounds, and known previous MRSA infections, and history of injection drug use. Three hundred patients were approached for participation; two-thirds of which met all study criteria and ultimately consented to participate in the study. All participants provided verbal informed consent. This study was approved by the Johns Hopkins Institutional Review Board.

### Collection of clinical specimens

Anterior nasal specimens were obtained using BBL Culture Swab Plus double applicators (Becton Dickinson, Sparks, MD) from 200 patients. Swabs were inserted into both left and right nostril of each study subject. One study coordinator collected all specimens.

### Analysis of clinical specimens

Swabs were inoculated into mannitol salts broth and allowed to sit for 5 minutes at room temperature. The swabs were then expressed into broth and incubated at 37°C for 4 hours. After this incubation, 300 μl of the sample was added to a tube containing zirconia beads and 200 μl of lysis buffer, and the sample was subjected to bead beating for 10 minutes (BioSpec Mini Bead Beater, Bartlesville, OK). Supernatants were transferred to a King Fisher 96-well processor and nucleic acids were isolated using the Ambion Total Nucleic acid (TNA) kit (Ambion, Austin, TX). Nucleic acids were analyzed in eight PCR reactions containing the duplex PCR primer pairs (Table 1) on PCR/ESI-MS platform (available for research applications only; Ibis Biosciences, Abbott, Carlsbad, CA). To determine the methicillin-resistance status of specimens from patients co-colonized with *S. aureus* and CoNS, samples were streaked on a ChromAgar (Becton Dickinson Biosciences, San Jose, CA) plate and incubated for 48 hours at 37°C.

**Table 1:** Primer pairs used in the PCR/ESI-MS MRSA typing and characterization assay

**Row**^**a**^	**Primer pair code**	**Target(s)**	**Accession number and coordinates**	**Forward and reverse primer sequences**	**Reference**
A	BCT879	*mecA*	Y14051	5′-TCAGGTACTGCTATCCACCCTCAA-3′	[14]
			4507..4581	5′-TGGATAGACGTCATATGAAGGTGTGCT-3′	
	BCT2157	*pta*	NC_003923	5′-TCTTGTTTATGCTGGTAAAGCAGATGG-3′	[13]
			629121..629229	5′-TGGTACACCTGGTTTCGTTTTGATGATTTGTA-3′	
B	BCT2249	*tufB*	NC_002758.2	5′-TGAACGTGGTCAAATCAAAGTTGGTGAAGA-3′	[14]
			615038..616222	5′-TGTCACCAGCTTCAGCGTAGTCTAATAA-3′	
	BCT2163	*yqi*	NC_003923	5′-TGAATTGCTGCTATGAAAGGTGGCTT-3′	[13]
			379057..379199	5′-TCGCCAGCTAGCACGATGTCATTTTC-3′	
C	BCT3016	*mupA*	X75439	5′-TAGATAATTGGGCTCTTTCTCGCTTAAAC-3′	[14]
			2482..2573	5′-TAATCTGGCTGCGGAAGTGAAAT-3′	
	BCT2161	*tpi*	NC_003923	5′-TCCCACGAAACAGATGAAGAAATTAACAAAAAAG-3′	[13]
			830671..830799	5′-TGGTACAACATCGTTAGCTTTACCACTTTCACG-3′	
D	BCT3106	TSST1	NC_002758.2	5′-TCGTCATCAGCTAACTCAAATACATGGA-3′	This work
			519..620	5′-TCACTTTGATATGTGGATCCGTCATTCA-3′	
	BCT3025	*arcC*	NC_003923	5′-TGAATAGTGATAGAACTGTAGGCACAATCGT-3′	[13]
			2724791..2724920	5′-TGCGCTAATTCTTCAACTTCTTCTTTCGT-3′	
E	BCT2095	PVL genes, *lukE-lukD*	NC_003923	5′-TGAGCTGCATCAACTGTATTGGATAG-3′	[14]
			1529595..1531285	5′-TGGAAAACTCATGAAATTAAAGTGAAAGGA-3′	
	BCT2149	*aroE*	NC_003923	5′-TGGGGCTTTAAATATTCCAATTGAAGATTTTCA-3′	[13]
			1674546..1674697	5′-TACCTGCATTAATCGCTTGTTCATCAA-3′	
F	BCT2256	*nuc*	NC_002758.2	5′-TACAAAGGTCAACCAATGACATTCAGACTA-3′	[14]
			894288..894974	5′-TAAATGCACTTGCTTCAGGGCCATAT-3′	
	BCT2166	*yqi*	NC_003923	5′-TAGCTGGCGGTATGGAGAATATGTCT-3′	[13]
			379190..379311	5′-TCCATCTGTTAAACCATCATATACCATGCTATC-3′	
G	BCT4022	PVL / l*ukD*	NC_003923	5′-TGGTCAACAAAGCTATATCAGTGAAGTAGAAC-3′	This work
			1529595..1531285	5′-TGGATCATGTCCAGACATTTCACCTAATG-3′	
	BCT2156	*gmk*	NC_003923	5′-TCACCTCCAAGTTTAGATCACTTGAGAGA-3′	[13]
			1191206..1191337	5′-TGGGACGTAATCGTATAAATTCATCATTTC-3′	
H	BCT4024	*ileS*	NC_002758.2	5′-TCAGTTGCTACAAGAGGAGTATCACCT-3′	This work
			1247700..1250453	5′-TACTCATCTTCTTACCTTCACCGTCCATA-3′	
	BCT2150	*aroE*	NC_003923	5′-TGATGGCAAGTGGATAGGGTATAATACAG-3′	[13]
			1674392..1674523′	5′-TAAGCAATACCTTTACTTGCACCACCTG-3′	

^a^Row indicates the row of a 96-well assay plate. Two primer pairs are present in each well of the assay plate.

### *Staphylococcus aureus* genotyping and characterization assay

**Figure 1:**
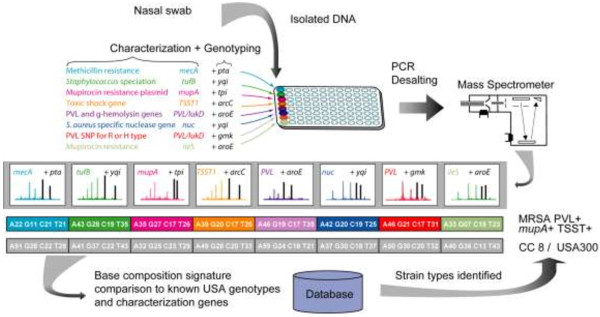
**Flow scheme for PCR/ESI-MS genotyping and characterization of *S. aureus*.** DNA from nasal swabs was distributed into eight wells of a microtiter plate, allowing 12 samples to be analyzed per plate. Each well contained two pairs of primers as indicated in Table 1. Following PCR and desalting, amplicons were analyzed by ESI-MS. Amplicon masses were used to calculate base compositions—the A, G, C, and T counts—of the PCR products. Comparison to a database of calculated base compositions of characterized strains allowed the determination of clonal complexes and USA types and of the presence or absence of genes for virulence factors, toxins and antibiotic resistance determinants.

In this study we combined elements from the previous genotyping [13] and characterization [14] assays into a single format and added primer pairs to interrogate the gene for toxic shock syndrome toxin 1 (TSST-1) [19], the PVL genes (distinguishing the “R” and “H” variants) [20] and the region of the *ileS* gene that encodes low level mupirocin resistance [21,22]. Thus, the current assay, (Figure 1), used eight amplification/mass spectrometry measurements to simultaneously detect the gene fragments useful for *Staphylococcus genotyping* and to unambiguously identify *S. aureus* and *S. lugdunensis* by distinguishing these species from each other as well as other coagulase-negative staphylococcal species. In addition, for *S. aureus* detections, the presence or absence of PVL (with a distinction between the “R” and “H” variants), the *mecA* gene, the gene encoding TSST-1, and the genes encoding high level (*mupA*) and low level (*ileS*) mupirocin resistance were determined along with the clonal complex. The turn-around time of the assay in this study, including the time for analysis was under 8 hours for the first 12 samples. Up to 100 samples can be analyzed in a 24 hours period.

### Risk factor evaluation

A structured nine-item questionnaire was designed based on review of the relevant literature, to identify patient demographic and other characteristics for hospital and/or community MRSA acquisition. Information collected included patient demographics (age, sex, race), a reason for visit to ED, whether they had a history of prescribed antibiotic, or history of admission to the hospital. Patient medical records were reviewed to provide clarification.

### Statistics

Prevalence estimates were calculated to estimate the proportion of emergency department patients with MRSA colonization. Identification of clinical features associated with MRSA colonization was initially assessed using bivariate analysis. Variables collected on the patient data sheet relating to MRSA colonization were entered into a bivariate logistic regression model to generate odds ratios and corresponding 95% confidence intervals of these factors, controlling for potential confounders.

## Results

### Genotype and characterization analyses

**Figure 2:**
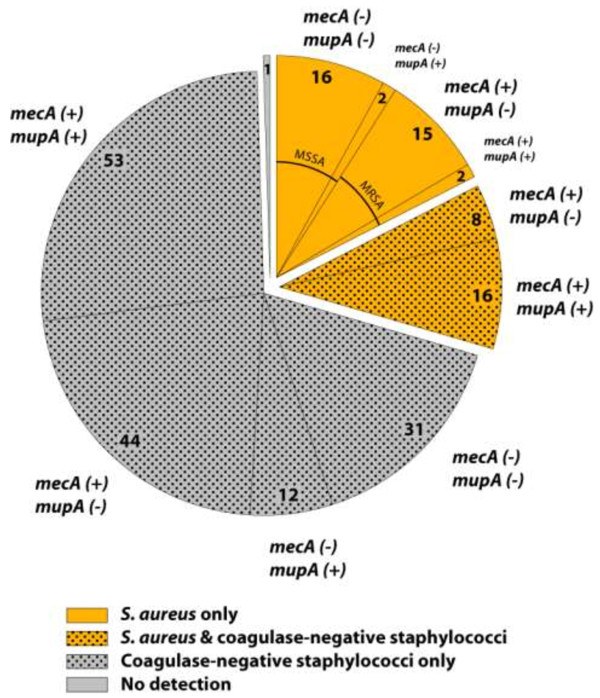
**Distribution of *S. aureus *(orange), coagulase negative staphylococci (stippled) and co-infections of both organisms (orange stippled) within a collection of nasal swabs from 200 patients.** Each category is subdivided according to the presence (+) or absence (-) of the mecA and mupA genes, with the indication of the corresponding number of isolates.

A total of 200 individuals were recruited and met eligibility criteria for this study. Demographic information for the 200 study participants is presented in Table 2. The results for PCR/ESI-MS analysis of nasal swabs from the participants are illustrated in Figure 2. The primer set used to analyze the nasal swabs contains a primer pair targeted to the highly conserved *tufB* gene that amplifies all species within the *Staphylococcus* genus but not other bacterial genera. Differences in masses of the amplicons in the mass spectra distinguish *S. aureus* and *S. lugdunensis* from all other species of staphylococci and identify *S. aureus* and CoNS. When both are present in a patient’s sample, spectral peaks from each species are observed in the same spectrum. Similarly, a primer pair targeted to the *gmk* gene, which is part of the genotyping component of the assay, also amplifies and distinguishes *S. aureus* and CoNS.

**Table 2:** Demographic characteristics of patients (n = 200) in the study

**Variable**	**All patients**	**SA carriers**	**MRSA carriers**
	**(200 subjects)**	**(59 subjects)**	**(27 subjects)**
**Age**			
18-29	54 (27%)	16 (27%)	7 (26%)
30-39	43 (21.5%)	15 (25.5%)	4 (15%)
40-49	55 (27.5%)	13 (22%)	9 (33%)
50-59	39 (19.5%)	13 (22%)	7 (26%)
60 and up	9 (4.5%)	2 (3.5)	0 (0%)
**Sex**			
Male	108 (54%)	26 (44%)	15 (55%)
Female	92 (46%)	33 (56%)	12 (45%)
**Race**			
White/Hispanic	38 (19%)	14 (24%)	4 (15%)
Black	159 (79.5%)	45 (76%)	23 (85%)
Asian, Pacific Islander	2 (1%)	0	0
American Indian Alaskan Native	0	0	0
Other	1 (0.5%)	0	0
**Hispanic/Spanish origin**			
Yes	3 (1.5%)	1 (2%)	0
No	195 (97.5%)	58 (98%)	27 (100%)
Unknown	2 (1%)	0	0
**Primary complaint**			
Respiratory	26 (13%)	6 (10.5%)	3 (11%)
GI	22 (11%)	7 (12%)	3 (11%)
Dermatology	5 (2.5%)	2 (3%)	1 (4%)
Localized pain/weakness	87 (43.5%)	20 (35.5%)	11 (41%)
UGI	7 (3.5%)	3 (4.5%)	1 (4%)
Injury	14 (7%)	4 (6%)	2 (7.5%)
Cardiovascular	19 (9.5%)	8 (13.5%)	3 (11%)
Other	18 (9%)	9 (15%)	3 (11%)
General pain/weakness	2 (1%)	0	0
**Antibiotic prescribed**			
Yes	39 (19%)	8 (14%)	5 (18.5%)
No	161 (79.5%)	49 (86%)	22 (71.5%)
**Admission to hospital**			
Yes	29 (14.5%)	13 (22%)	7 (26%)
No	171 (75.5%)	46 (78%)	20 (74%)

Of 200 patients presenting to the emergency department, 59 (29.5%) were colonized with *S. aureus*; 24 of these patients were also co-colonized with CoNS (Figure 2)*.* One hundred forty of the 200 (70%) were colonized exclusively with CoNS. Only one patient showed no evidence of staphylococcal colonization.

The distribution of the *mecA* gene (the methicillin resistance gene) and the *mupA* gene (responsible for high level mupirocin resistance) among the detected Staphylococcus species is also reported in Figure 2. Of 35 patients colonized only with *S. aureus*, 18 were methicillin-sensitive *S. aureus* (MSSA) and 17 were MRSA. Of these 18 MSSA patients, 11.1% (2/18) were colonized with strains that carried the gene for high-level mupirocin resistance. Of 17 MRSA isolates, 11.8% (2/17) had the mupirocin resistance gene. Of 140 CoNS positive patients, 8.6% (12/140) were colonized with strains that had only the mupirocin resistance gene, 31.4% (44/140) had only methicillin resistance genes and 37.9% (53/140) had genes encoding both methicillin and mupirocin resistance.

Among the 24 patients who were co-colonized, 66.7% (16/24) had colonies with both methicillin and high-level mupirocin resistance, while 33.3% (8/24) had methicillin resistance only. In those co-colonizations, the location of the antibiotic resistance genes could not be determined directly by PCR/ESI-MS (as the same amplicon is produced irrespective of the bacterial strain); instead, the species carrying the gene was determined by plating samples on ChromAgar in order to demonstrate more than one colony type. Since in ten instances methicillin resistance could thus be attributed to *S. aureus*, 45.8% (27/59) of patients colonized with *S. aureus* were indeed colonized with MRSA. Also, 85 patients altogether were colonized with strains that carried the gene encoding high-level mupirocin resistance.

The genotypes and presence of virulence factors unique to *S. aureus* were determined for all patients colonized with *S. aureus*, regardless of whether the patient was co-colonized with CoNS. The genotype and characterization results are shown in Table 3. The 59 *S. aureus* isolates showed a remarkable diversity; the isolates clustered into 21 genotypes as depicted by each unique row in Table 3. One genotype was detected in 12 instances (Table 3) in this patient population, but 11 patients had unique genotypes. When examined from the perspective of clonal complexes, the 21 unique molecular genotypes belong to seven major clonal complexes (CC) (Table 3). The most abundant genotypes were PCR/ESI-MS type 1 (CC8) (19 patients), which correlates with USA300, followed by PCR/ESI-MS type 2 (CC5), which correlates with USA100 (12 patients). Eleven patients had genotypes that were PCR/ESI-MS type 1 and were positive for the PVL “R” variant; these genotypes are consistent with the predominant community-associated MRSA (CA-MRSA) in the US [20]. PCR/ESI-MS type 2 (CC5) was isolated from 12 patients; these isolates were PVL negative and these organisms are consistent with hospital-acquired MRSA (HA-MRSA). PCR/ESI-MS types 1 and 2 (CC8 and CC5) represented slightly more than half the organisms isolated from this patient population; the remainders represented a plurality of CC genotypes (CC45, CC30, CC15, CC45, CC59 or CC1) or did not fit into a previously defined clonal complex (Table 3).

**Table 3:** **Determination of PCR/ESI-MS type and clonal complexes of *S. aureus *** **from 59 infected patients**^**1**^

**Genotype**		**Virulence/Toxin-encoding genes**				**Number of instances**
*Clonal Complex*	*PCR/ESI-MS or ST Type*^*2*^	*LukD/E*	*PVL*	*PVL Type*	*TSST*	
CC8	1	-	-	-	-	1
		lukD/E+	-	-	-	6
		lukD/E+	-	-	TSST+	1
		lukD/E+	PVL+	R	-	11
	7	lukD/E+	-	-	-	1
CC5	2	lukD/E+	-	-	-	12
CC30	3	-	-	-	-	2
		-	-	-	TSST+	2
CC15	12	lukD/E+	-	-	-	4
CC45	6	lukD/E+	PVL+	R	-	1
		-	-	-	TSST+	1
		-	-	-	-	3
CC59	5	-	-	-	-	3
CC1	11	lukD/E+	-	-	-	1
	ST:20 or ST:389	lukD/E+	-	-	-	3
	ST:115, ST:116, or ST:118	-	-	-	-	2
	ST:115	-	-	-	-	1
	ST:25, ST:28, ST:564, ST:297 or ST369	lukD/E+	-	-	-	1
	ST:109 or ST:540	-	-	-	-	1
	ST:630 or ST:683	-	-	-	-	1
	ST:188, ST:663 orST:758	-	-	-	-	1

^1^PCR/ESI-MS type and clonal complex correlated as previously described [2].

^2^In cases where the PCR/ESI-MS was not conclusive the best match consistent with ST type is shown.

Additional molecular resolution beyond the PCR/ESI-MS type was provided by the presence or absence of the *lukD/E* genes and the gene encoding the TSST-1 toxin. Regions of two leukocidin toxin-producing operons were amplified by a single pair of primers, BCT2095. A region of the Panton-Valentine leukocidin (PVL) operon, which includes the *lukS-PV* and *lukF-PV* genes, is targeted by a primer pair that hybridizes across the junction of the 3′ end of *lukS-PV* and the 5′ end of *lukF-PV*[14]. The same primer pair serendipitously detects and differentiates by virtue of base composition a second leukotoxin-encoding operon that contains the *lukE* and *lukD* genes. Thus, analysis of an isolate had four potential outcomes: PVL positive, *luk*E/D positive, both positive, or neither positive. The *lukD/E* genes are generally correlated with the clonal complex assignment. It was recently shown that the gene encoding TSST-1 protein was associated with PFGE type USA200 in 87% of isolates studied [23]. The *tst* gene was present in two of the four instances of this genotype in our study. No mutations associated with low-level mupirocin resistance were observed in the *ileS* genes within this data set.

### Risk factor analysis for colonization

Patient demographics, primary complaints, antibiotic prescriptions and providers’ admission decisions were evaluated for association with particular pathogens. No independent factor was associated with any type of colonization, however African Americans were more likely to be colonized with USA 100/300 *S. aureus* genotypes than other races (28 (62%) versus 3 (21%), p = 0.013).

## Discussion

While some Emergency Departments have a high prevalence of MRSA isolated from patients with sporadic skin and soft-tissue infections [24-26], limited research has explored nasal MRSA carriage in this population. The mechanisms leading to *S. aureus* nasal carriage are multifactorial, and an optimal fit between host and bacteria seems to be essential [27]. This study demonstrated a significant prevalence of *S. aureus*, MRSA and drug-resistant CoNS nasal colonization in a random sample of individuals seeking care from the Emergency Department at The Johns Hopkins Hospital. The prevalence of MRSA (13.5%) among nasal isolates was somewhat greater than those estimated from other studies that had a similar objective and sample population [28]. Three studies performed outside healthcare facilities (*i.e.*, isolated, semi-closed rural population [29], urban poor in San Francisco [30], or university students in Portugal [31]) that include a total of 4,452 people, showed a pooled MRSA colonization prevalence of only 0.76% [32]. The prevalence of MRSA colonization in an inpatient setting in a tertiary hospital was reported to be 0.18%–7.2% [32,33]. Our findings were similar to the prevalence noted in a study investigating the prevalence of MRSA among newly arrested men in Baltimore (29.5% and 13.5% in our study vs. 40.4% and 15.8% for *S. aureus* and MRSA prevalence, respectively) [34]. Other studies have identified three risk factors for MRSA colonization: hospitalization within the past 12 months [16], previous MRSA infection [17] and injection drug use [18]. Because these risk factors had already been identified, they were exclusion criteria in our study. Although we evaluated patient demographics, primary complaints, chronic co-morbidities, antibiotic prescriptions and providers’ admission decisions, none were independent risk factors for MRSA colonization.

Our data confirms recent epidemiologic data indicating that nosocomial MRSA strains are present in the community. Interestingly, there was substantial genotypic diversity of *S. aureus* and MRSA types in this the patient population. There was a significant frequency of co-colonization of *S. aureus* and CoNS (24 patients, 12%) and at least one third of patients were colonized with a CoNS that had a high-level mupirocin resistance gene (65 patients or 32%, non- counting the *S. aureus* co-infections). This is concerning because it has previously been reported that the gene for high-level mupirocin resistance can be found on conjugative plasmids that carry multiple resistance determinants for other antimicrobial agents [21]. Moreover, this resistance has been shown to be transferable to *S. aureus*[35], which has implications for mupirocin decolonization strategies.

The PCR/ESI-MS methodology operates on an automated, high-throughput platform; patient specimens are screened without culture and results are obtained in a time frame similar to existing molecular tests. As data from more extensive analysis of the *S. aureus* strains that result in the hospital infections become available, additional targets for genetic analysis of *S. aureus* can be added. While a goal of this assay was to add targets that provide information that have value for infection control and tracking outbreaks in hospital settings, the limitation to a current assay is the lack of resolution compared to other technologies. We acknowledge that the inclusion of typing for TSST or PVL, would probably not aid in a clinical scenario, but might aid a research study where predictors of subsequent infection in hospitalized patients were being studied.

This study was performed in one location, the Johns Hopkins Hospital Emergency Department and, therefore, these results may not be applicable to other areas of the United States. Although the ethnic diversity of the study participants was similar to that of the population of Johns Hopkins Hospital patients, of which 80% are African American, these results may not be applicable to more ethnically diverse areas of the United States. It also should be noted that literature cites significant heterogeneity in the risk factors for nasal *S. aureus* and MRSA colonization, as well as the fact that only surveying nasal swabs likely underestimates the prevalence of S. aureus and MRSA carriage [4,36]. Several other, previously described risk factors for *S. aureus* and MRSA colonization (i.e. previous admission to hospital, or previous antibiotic use) were also investigated in this study. However, there are other known factors for which we do not have data, that might have significantly contributed to the relatively high burden of MRSA encountered in our study, including HIV status, diabetes, men who have sex with men, recent incarceration, high-risk sexual behavior, household members with SSTI, and hospital-associated exposures [4]. It should be noted that this was not a longitudinal study that would distinguish at least three *S aureus* nasal carriage patterns in healthy individuals: persistent carriage, intermittent carriage, and non-carriage. This distinction is important because persistent carriers have higher *S. aureus* loads and a higher risk of acquiring *S. aureus* infection [37,38]. A further important limitation is that in the significant proportion of patients (~40%) with both *S. aureus* and CNS, this assay alone cannot distinguish for the presence of MRSA or other resistance determinants being carried by *S. aureus*. Thus, there remains the need for plating samples on ChromAgar, which adds significantly to the turn-around time for infection control purposes.

## Conclusions

In conclusion, the prevalence of *S. aureus* and MRSA colonization in a convenience sample of patients seeking care in Emergency Department was 29.5% and 13.5%, respectively. A substantial fraction of the *S. aureus*-colonized patients were co-colonized with CoNS and high-level mupirocin-resistant CoNS. These findings highlight the importance of active surveillance studies to detect MRSA colonization and/or infection among emergency department patients. The molecular genotypes were directly identified from nasal swabs by PCR/ESI-MS. The ability to identify unique molecular signatures from MRSA may allow infection control resources to be effectively focused and will enable tracking origins of outbreaks in hospital settings. Determining the molecular genotype of *S. aureus* during intake screening may prove valuable if certain molecular genotypes become associated with increased infection risk.

## Abbreviations

PCR/ESI-MS: Polymerase chain reaction to mass spectrometry; CoNS: Coagulase-negative staphylococci; MRSA: Methicillin-resistant *Staphylococcus aureus*; MLST: Multilocus sequence typing; PFGE: Pulsed-field gel electrophoresis; MSSA: Methicillin-sensitive *S. aureus*; CC: Clonal complex; CA-MRSA: Community-associated MRSA; HA-MRSA: Hospital-acquired MRSA.

## Competing interests

Charlotte Gaydos has received funds for speaking and travel from Abbott. Charlotte Gaydos and Richard Rothman have received research funding from Abbott. Ray Ranken, David J. Ecker, Christian Massire, Rangarajan Sampath, and Lawrence B. Blyn are employees of Abbott.

## Authors’ contributions

AK, CG, RR, DJE, RS, and LBB conceived of the study, and participated in its design and coordination and helped to draft the manuscript. RR and CM carried out the genotyping and characterization assay and drafted the manuscript. Y-HH participated in the design of the study and performed the statistical analysis. All authors read and approved the final manuscript.

## Pre-publication history

The pre-publication history for this paper can be accessed here:

http://www.biomedcentral.com/1471-2334/14/16/prepub
